# Integrated focal psychotherapy: Results from a retrospective study

**DOI:** 10.3389/fpsyg.2023.945644

**Published:** 2023-02-13

**Authors:** Maria Clotilde Gislon, Davide Sattin, Mattia Cis, Mara Fiaschi, Giacomo Tognasso, Vincenzo D’Ambrosio, Maria Villa, Milena Ruffino, Susanna Bergamaschi

**Affiliations:** ^1^Institute for the Study and Research of Psychic Disorders (ISeRDiP), Milan, Italy; ^2^Istituti Clinici Scientifici Maugeri IRCCS, Milan, Italy

**Keywords:** integrated psychotherapy, focal, retrospective study, cognitive treatment, psychodynamic, outcome assessment, CORE-OM, cognitive rehabilitation

## Abstract

**Background:**

The focus-based integrated model (FBIM) is a form of psychotherapy that integrates psychodynamic and cognitive psychotherapy and Erikson’s life cycle model. Although there are many studies on the effectiveness of integrated models of psychotherapy, few have examined the efficacy of FBIM.

**Objective:**

This pilot study explores clinical outcome measures concerning individual wellbeing, the presence/absence of symptoms, life functioning, and risk in a cohort of subjects after they received FBIM therapy.

**Methods:**

A total of 71 participants were enrolled at the CRF Zapparoli Center in Milan, 66.2% of whom were women (*N* = 47). The mean age of the total sample was 35.2 years (SD = 12.8). We used the Clinical Outcomes in Routine Evaluation–Outcome Measure (CORE-OM) to test treatment efficacy.

**Results:**

The results revealed that participants improved in all four dimensions of CORE-OM (i.e., wellbeing, symptoms, life functioning, and risk), women improved more than men, and in most cases (64%), the change was clinically reliable.

**Conclusion:**

The FBIM model seems to be effective for treating several patients. Most of the participants saw significant changes in symptoms, life functioning, and general wellbeing.

## 1. Introduction

Integrated psychotherapy, which has become increasingly popular in recent years, has a long history. It emerged in the 1930s when the first attempts were made to integrate Freud’s and Pavlov’s theories (French, 1933). In the 1950s, Donald and Miller proposed modifications to psychoanalytic practice by introducing therapeutic modalities typical of a behavioral approach (such as a more active role for the therapist, giving homework to patients, role-playing, and modeling) to address symptoms. Attempts to integrate different methods continued in the 1960s and 1970s. *Psychoanalysis and Behavior Therapy* (Wachtel, 1977) was the first manual to propose a clinically and theoretically structured integration of dynamic and behavioral theories. The debate on the advantages and limits of integrating cognitive and psychodynamic theories continued in the 1980s and 1990s (Arkowitz, 1992).

Nowadays, most patients show a combination of symptoms and relational problems, but often, psychotherapies fail to treat both (Gazzillo et al., 2021) or focus on the former at the expense of the latter (Nordahl et al., 2018). By contrast, psychodynamic approaches are more centered on relationship dynamics rather than cognitive and behavioral techniques that decrease symptomatology (Stolorow et al., 1987; Cain, 2002; Markowitz and Weissman, 2004). Moreover, according to Gazzillo et al. (2021), many psychotherapies involve a top-down approach, are based on manuals that recommend specific methods and techniques to the patient and are predicated on preordained diagnoses. Meanwhile, the bottom-up approach is case-specific and based on a comprehensive diagnosis of the patient’s needs (Silberschatz, 2017). Many researchers (Dimaggio, 2015; Gazzillo et al., 2021; Suzuki et al., 2021) argue that psychotherapy has to be customized, and this means paying close attention to the functional diagnosis process. Integrated psychotherapy recognizes the patient’s needs and manages their symptomatology (Zapparoli, 2009).

The literature on integrated psychotherapies has grown considerably (Feldman and Feldman, 2005). Several studies have demonstrated the effectiveness of integrated approaches (Beutler et al., 2012; Miscioscia et al., 2018). In Schottenbauer et al.’s (2005) review, nine studies showed substantial support, 13, some support, and seven, preliminary support.

Integration generally takes three paths: (a) a mixed-use of techniques without reference to a particular model; (b) a specific model of techniques of varying orientations; or (c) a combination of theory and practice that lead to the formation of a new model deriving from two initial psychotherapeutic models (Castonguay et al., 2015).

### 1.1. The FBIM model

For the last decade, the Institute for Study and Research on Mental Disorders (ISeRDiP) has been developing the focus-based integrated model (FBIM; Gislon, 2000), a new approach to psychological wellbeing (Gislon, 2005). It offers effective and brief treatment for people affected by psychological disorders, an appropriate response to associated emergencies, and a prevention and health enhancement program (Zapparoli, 2002). Treatment planning encompasses a range of integrative psychotherapeutic strategies and techniques (e.g., psychoanalytic, cognitive–behavioral, and developmental or life cycle models and findings from research on resilience) and other interventions (e.g., pharmacotherapy, social interventions, and rehabilitation for severe mental diseases).

The FBIM, which is based primarily on focal interventions, integrates psychodynamic psychotherapy of Freudian origin (Ellenberger, 1996) with Erikson’s life cycle model and cognitive–behavioral psychotherapy (D’Ambrosio et al., 2006). The average duration of treatment is fewer than 40 sessions.

Integrated focal therapy is applied to all diagnoses, but the objectives may be different. Those for the most severe cases include a reduction in dysfunctionality and the integration of prostheses to rehabilitate functions and improve the quality of life (Zapparoli and Scortecci, 1988). By contrast, the targets for less severe cases include unblocking the patient’s central intrapsychic conflict and allowing it to resume its life cycle.

It is essential to identify where the patient is in the life cycle and any developmental tasks that are challenging them (Gislon, 2005). These tasks may arise from internal pressures, for example, sexual needs in adolescence or the need for stability in an adult partnership, or external events that disrupt their sense of security (e.g., the loss of a job or a medical diagnosis). Some individuals do not have the resilience to respond to change because they have developed excessive fears and defend themselves against these in a dysfunctional way, that is, by putting up defenses that block their own evolution (Gislon, 2000). Such defenses are inconsistent with the patient’s needs and may generate symptoms based on self-deception (Lingiardi et al., 2011). They then form the basis of the patient’s new functioning, which is the *focus* of our specific diagnosis. The question we use to find this focus is as follows: “What the patient is afraid of, and how do they defend themself against it?” (Gislon, 2005). Sometimes, these defenses are embedded within a conflict between the desire to satisfy a need and the fear that this entails. Sometimes they are pervasive and based on a desire to be omnipotent.

The FBIM has four fundamental pillars: (a) a specific diagnosis; (b) the focus; (c) the integrated intervention; and (d) setting the patient up for self-therapy (Gislon, 2005).

#### 1.1.1. The diagnosis

In the first three/four sessions, the FBIM therapist makes a nosographic and specific functional diagnosis. In the less severe cases, the therapist identifies the developmental dilemma and conflict; the latter is an essential change agent involving an internal dialogue between opposing cognitive and emotional schema (Zapparoli and Scortecci, 1988).

#### 1.1.2. The focus

The first part of the psychotherapeutic process is dedicated to explaining the focus of the intervention to the patient. This phase is the core of the intervention. The focus corresponds to the central conflict hindering the patient and the natural course of his/her life cycle (Gislon, 2000). The goal is to use the patient’s narrative to show them how their major conflict is created, what role it serves, the fear underpinning it, and the dysfunctional nature of the defense against it.

#### 1.1.3. The integrated intervention

The intervention is customized for each patient. In most cases, it involves cognitive modules (Sinibaldi, 2009) aimed at symptoms and psychodynamic interventions and working on the fundamental intrapsychic conflict (Villa, 2011). The therapist also acts as an intermediary between the dysfunctional modalities of patients and their resources, helping them solve the conflict and progress toward a more constructive and adaptive perspective. The patient’s goal is to find new tools to cope with their symptoms and understand the intrapsychic motivations that have interfered with their life and the fulfillment of their needs.

#### 1.1.4. Self-therapy

Once the patient has internalized the awareness of their central functioning, they have permanently experienced a new perspective and internalized the tools to manage the symptoms. They are now set up for self-therapy. In this phase, the therapist sees the patient less frequently and supervises the work that the patient, who has become an expert, does on themself (Gislon, 2000).

The FBIM model considers the patient’s *potential to change*, their *security system*, and *resistance to change* when making a functional diagnosis.

*The potential to change* is identified by: (a) personality structure and self-concept, defense and coping mechanisms (from a primitive to higher to mature level), tolerance dependence, frustration, loss, and the capacity for self-regulation; and (b) the developmental process and its dimensions: cognitive functioning, sexual functioning, and relational modalities; narcissism and self-esteem; and the capacity for self-observation and self-knowledge. Each person is endowed with evolutionary potential, that is, adaptation and resilience, the ability to find a balance between activeness or passivity in meeting one’s own needs, the capacity to accept limits, and the possibility of self-observation or insight (Gislon, 2005).

According to Erikson’s (1950) model, the security system is connected with the idea that the person is inserted in an evolutionary context. They are ordinarily resilient in the face of developmental tasks that require adaptation. Their predominantly external security system becomes more internal as they grow up. This system allows them to tolerate fear and anguish, accept the limits that life imposes, and exploit their evolutionary potential to cope with reality. The system may be realistic or unrealistic and, as such, based on self-deception.

#### 1.1.5. Resistance to change

During the therapy, mental resistance to change may emerge. The patient’s defenses may be overly rigid, and they may find it difficult to accept help.

The principal aim of the present study was to explore the clinical outcomes of a pilot study involving a sample of patients progressively enrolled and treated using the FBIM. In particular, we wanted to assess possible changes in four dimensions: (a) individual wellbeing; (b) symptoms; (c) general life functioning; and (d) risk of self-harm in accordance with Clinical Outcomes in Routine Evaluation–Outcome Measure (CORE-OM; Evans et al., 2000).

## 2. Materials and methods

### 2.1. Participants

Our sample consisted of 71 participants recruited from the CRF Zapparoli Center in Milan, 33.8% were men (*N* = 24), and 66.2% were women (*N* = 47). All patients were new to the Center. All participants, who were aged between 18 and 65 (average 35.2, SD = 12.8), were of Italian origin. Patients with psychotic symptoms (e.g., delusions and hallucinations) were excluded from the study; such individuals were referred to specialized centers. Educational levels ranged from high school to graduate, the youngest were single or engaged, and some older participants were married. All these data have not been described in the table or included in the analyses because we started from the assumption that they are not impediments to successful psychotherapeutic outcomes (Table 1). For details of the focus distribution and International Statistical Classification of Diseases and Related Health Problems, the 10th revision (ICD-10) diagnoses, see Table 2. All the therapists involved in the study were specialists from IseRDiP, and all had 4 years of experience in clinical settings.

**TABLE 1:** Demographic and clinical data of the sample (*n* = 71).

Variables	*N*	%
**Gender**		
Male	34	33.8
Female	47	66.2
	**Mean**	**SD**
Age	35.2	12.8
Sessions	38.7	14.6

**TABLE 2:** Focus distribution according to ICD diagnosis.

	Focus								
	**F1**	**F2**	**F3**	**F4**	**F5**	**F6**	**F7**	**F8**	
**ICD × diagnosis**									
F30–39	2	0	0	0	1	0	2	3	8
	25.0%	0%	0%	0%	12.5%	0%	25.0%	37.5%	100%
F40–49	5	1	5	1	1	12	9	3	37
	13.5%	2.7%	13.5%	2.7%	2.7%	32.4%	24.3%	8.1%	100%
F50–59	2	0	0	0	0	3	1	0	6
	33.3%	0%	0%	0.0%	0%	50.0%	16.7%	0%	100%
F60–69	4	0	9	0	2	2	1	2	20
	20.0%	0%	45.0%	0%	10.0%	10.0%	5.0%	10.0%	100%
	13	1	14	1	4	17	13	8	71
	18.3%	1.4%	19.7%	1.4%	5.6%	23.9%	18.3%	11.3%	100.0%

F30–F39—mood affective disorders; F40–F49—anxiety, dissociative, stress-related, somatoform, and other non-psychotic mental disorders; F50–F59—behavioral syndromes associated with physiological disturbances and physical factors; F60–F69—disorders of adult personality and behavior. F1 = Conflict between recognition and non-recognition of one’s own needs. F2 = Conflict between narcissistic position and reciprocity. F3 = Conflict between destructive and constructive aggression. F4 = Conflict between pain and pleasure. F5 = Conflict between real and ideal self-image. F6 = Conflict between omnipotence and limit. F7 = Conflict between dependence and autonomy. F8 = Conflict between compensation and forgiveness.

### 2.2. Measures

#### 2.2.1. Outcome measure

We used the CORE-OM (Evans et al., 2000; Italian version: Palmieri et al., 2009) to assess treatment outcomes. The CORE-OM, which comprises 34 items, is a self-report measure widely used in clinical practice. It is reliable, valid, and acceptable in a broader range of settings (Barkham et al., 2001; Evans et al., 2002; Shepherd et al., 2005). Items include a 5-point Likert-type scale (0 = *not at all*; 4 = *most of or all the time*) and cover four domains: (a) subjective wellbeing (four items); (b) problems/symptoms (12 items); (c) life functioning (12 items); and (d) risk (six items). Higher scores on all domains suggest severe problems by reversing scoring on eight positively keyed items; total scores have been reported as the mean across completed items. The CORE-OM scores exhibited good internal consistency in this sample, as Cronbach’s alpha varied from 0.86 at baseline to 0.93 at the end of the treatment in the clinical group.

#### 2.2.2. Focus identification

An *ad hoc* questionnaire was used to identify the different foci (of which there were eight) for each participant. The therapist was required to choose the correct one.

*Focus 1* (F1): Recognizing one’s own needs. The patient is afraid about the nature of their needs or the consequences of searching for the object of their needs; typically, the patient defends themself by not feeling their needs or not finding adequate objects of need.

*Focus 2* (F2): Conflict between narcissistic position and reciprocity. The patient does not tolerate the limits imposed by others and considers a relationship to be the single way of satisfying their needs.

*Focus 3* (F3): Conflict between destructive and constructive aggression. The patient experiences aggressiveness as dangerous and defends themself by not expressing it or turning it against them.

*Focus 4* (F4): Conflict between pain and pleasure. The patient is afraid of feeling pleasure because they cannot tolerate not having control over its consequences. In such cases, patients usually defend themselves by holding onto the pain because they can control it.

*Focus 5* (F5): Conflict between real and ideal self-image. The patient is afraid of being worthless or not being up to par; they defend themself by creating an ideal self-image.

*Focus 6* (F6): Conflict between omnipotence and limits. The patient experiences limits as the enemy because these create anguish, sadness, or anger; they respond by attempting to become omnipotent.

*Focus 7* (F7): Conflict between dependence and autonomy. The patient is afraid of the need for autonomy that arises at different stages in the evolutionary process and defends themself by remaining dependent on old external securities.

*Focus 8* (F8): Conflict between compensation and forgiveness. The patient is afraid of being damaged by objects of need. They defend themself by waiting for external compensation.

#### 2.2.3. Procedure

Our sample comprised 71 participants recruited at the CRF Zapparoli Center over 2 years (2017 and 2018). They were diagnosed by independent psychiatrists. Diagnoses ranged from less severe (i.e., mood, anxiety, stress-related, somatoform, and other non-psychotic disorders) to more severe (i.e., addiction problems, eating disorders, and personality disorders). After being diagnosed, the participants were referred to individual psychotherapists who decided how to structure the therapy based on the functional diagnosis carried out during the first sessions. The total number of sessions depended on the severity of the individual patient’s symptoms and the focus.

Before the participants agreed to participate in the research, a research assistant briefly explained the content and the purpose of the study. All collected questionnaires were anonymous, and the patients were informed that they could withdraw from the study anytime. The CORE-OM was administered in two sessions (at the beginning [T0] and at the end [T1] of therapy) after authorization to use personal data was received by the Italian privacy law no. 675/96. Written informed consent was obtained from the participants before they filled in the questionnaires, which were subsequently codified digitally and scored based on the instructions provided by the scale’s author. The study received no grants from funding agencies in the public, commercial, or non-profit sectors.

### 2.3. Statistical analysis

Because the study was exploratory, data were first analyzed descriptively. Means, standard deviation (SDs), and confidence intervals (CIs) were reported for continuous variables and integers and percentages for the categorical ones.

We expected to observe a difference in the CORE-OM scales, so the differences between pre- to post-treatment were calculated through paired *t-*tests or the Wilcoxon–Mann–Whitney test according to normal or non-normal data distribution. In view of the moderate sample size and the explorative aim of the study, we applied the Bonferroni correction to limit type 1 error; the effect size was calculated wherever possible.

The reliability and clinical significance of the changes were assessed using the Jacobson and Truax (1991) criteria. These can be used to measure change at the level of the patient and are especially useful for small-sample studies, where group variance may mask individual changes. The method comprises two steps. The first calculates the reliable change index (RCI) from a function of the remainder of the post- to pre-test, the initial standard deviation of the measure, and its reliability:


$$R\,C\,I=x_{\text{post}}-x_{\text{pre}}/S\,d\,i\,f\,f,$$

where Sdiff = √2(SE)2 and SE = S1√1-reliability, and S1 is the normative standard deviation at baseline and the internal consistency value (alpha). The reference values were ±1.96 to determine improvement, no change, or deterioration (> +1.96, < +1.96, and >−1.96 and <−1.96, respectively). The cut-off clinical (C) value, which indicated a weighted midpoint between the means for the patient and non-patient population, was set to 1.0 for the entire sample in the total score according to Italian normative data (Palmieri et al., 2009) and Evans et al.’s (2002) methodology.

A box plot was developed to show pre–post scores and outliers according to the clinical change in each patient. The sample was then divided to reflect clinical and reliable changes: (a) significant/reliable improvement; (b) no change; and (c) deterioration. Data were then represented in graphic and tabular form, including those for participants for whom the clinically significant change was not associated with a reliable change and vice versa. Participants whose baseline scores were under the cut-off C value were not categorized in this way. Effect sizes were calculated using Gpower^®^ software (Faul et al., 2007, 2009), with their values classified as small (*d* = 0.2), medium (*d* = 0.5), and large (*d* ≥ 0.8).

Statistical significance was set at *p* < 0.05 for all the analysis, which was performed using SPSS Version 24.0 (IBM Corporation, 2016).

## 3. Results

The majority of the participants were women (66%; *N* = 47), and the mean age of the total sample was 35.2 (SD = 12.8). The two most prevalent ICD diagnoses were those associated with anxiety (F40−F49) and personality and behavior disorders (F60−F69). The mean total number of sessions was less than 40, with a relatively stable mean focus distribution of 40–50 in most subgroups (F1 *M* = 43 SD = 20, F3 *M* = 40 SD = 10, F5 *M* = 49 SD = 25, F6 *M* = 43 SD = 10, F7 *M* = 31 SD = 12, F8 *M* = 28, SD = 9). Focus groups 2 and 4 comprised only one patient each. The most common baseline disorders were anxiety, dissociative, stress-related, somatoform, and other non-psychotic mental; adult personality; and behavior conditions.

Two missing data items were reported for one patient in the CORE-OM test at baseline during the outcome measure analysis, so their information was excluded. Of the remaining 70 patients, five showed a CORE-OM total score under the cut-off value of 1 at baseline, so they were considered a non-clinical sample, though they were treated (the mean number of treatment sessions for the non-clinical sample = 34, SD = 9.8 vs. a mean session number = 39, SD = 14.8 for the clinical group). No correlations were found between CORE-OM pre–post scores and the number of sessions. Table 3 shows both non-clinical and clinical group data for all the CORE-OM subscales. Paired *t*-tests revealed a statistically significant difference for all the scores, with a large effect size for almost all scores (except the risk scale). Differences between men (*n* = 21) and women (*n* = 44) were found in the CORE-OM total score at baseline (*U* = 638, *p* = 0.014, effect size = 0.68) in the clinical group, with the women showing higher values than the men.

**TABLE 3:** Clinical Outcomes in Routine Evaluation–Outcome Measure (CORE-OM) scores distribution pre- and post-therapy.

	Non-clinical sample (*n* = 5)			Clinical sample (*n* = 65)					
**CORE-OM**	**Pre-therapy score** **mean (SD)**	**Post-therapy score** **mean (SD)**	**Mean Δ** **(post-pre)** **mean (CI)**	**Pre-therapy score** **mean (SD)**	**Post-therapy score** **mean (SD)**	**Mean Δ** **(post-pre)** **mean (CI)**	***t***	**Effect size**	***P*-value**
Wellbeing	1.3 (1.0)	0.5 (0.4)	−0.8 (−1.7/0.2)	2.7 (0.7)	1.1 (0.7)	−1.7 (−1.9/−1.5)	16.0	1.8	0.000
Symptoms	1.1 (0.6)	0.4 (0.1)	−0.7 (−1.3/0.0)	2.4 (0.6)	0.9 (0.6)	−1.5 (−1.8/−1.4)	15.4	1.8	0.000
Soc fun	1.3 (0.7)	0.4 (0.2)	−0.8 (−1.8/0.2)	2.1 (0.6)	0.9 (0.5)	−1.2 (−1.4/−1.0)	13.3	1.7	0.000
Risk	0.2 (0.4)	0.0 (0.1)	−0.1 (−0.5/0.2)	0.3 (0.5)	0.0 (0.2)	−0.2 (−0.4/−0.1)	3.8	0.6	0.000
Total – risk scale	1.3 (0.9)	0.4 (0.1)	−0.9 (−2.0/0.2)	2.3 (0.5)	0.9 (0.5)	−1.4 (−1.5/−1.2)	15.8	2.0	0.000
Total score	0.6 (0.3)	0.3 (0.1)	−0.2 (−0.7/0.3)	2.0 (0.5)	0.8 (0.4)	−1.1 (−1.3/−1.0)	15.3	1.9	0.000

**FIGURE 1:**
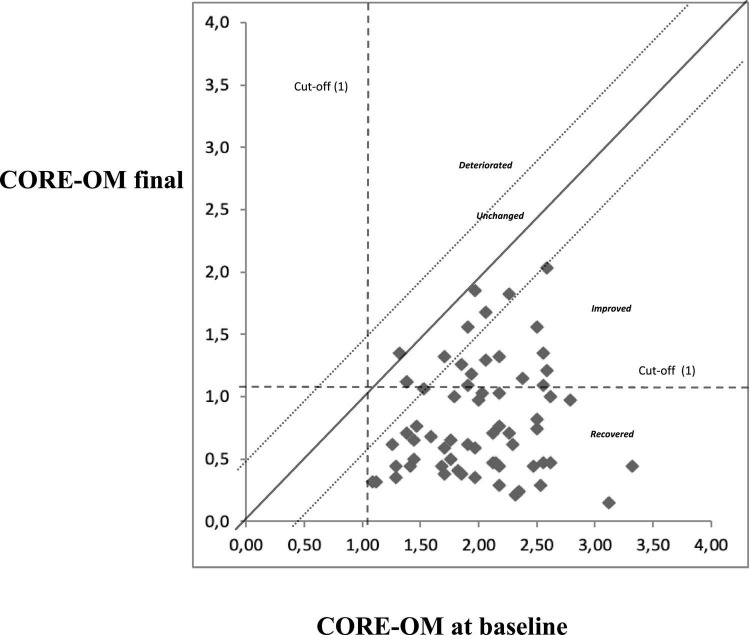
Graphical representation of changes in Clinical Outcomes in Routine Evaluation–Outcome Measure (CORE-OM) total score between baseline and end of treatment.

No clinical or statistical deterioration was found in the clinical sample after treatment. A total of 41 (63%) patients reported a clinical improvement after the treatment, and 42 (64%) showed a reliable change (Figure 1 and Table 4).

**TABLE 4:** Reliable and clinically significant change of the clinical sample at baseline.

Clinically significant change	Reliable change			
	**Reliable deterioration**	**No reliable change**	**Reliable improvement**	**Total**
Clinically significant improvement	0	7 (10.8%)	34 (52.3%)	41 (63.1%)
No clinically significant change	0	16 (24.6%)	8 (12.3%)	24 (36.9%)
Clinically significant deterioration	0	0	0	
Total		23 (35.4%)	42 (64.6%)	65 (100%)

**FIGURE 2:**
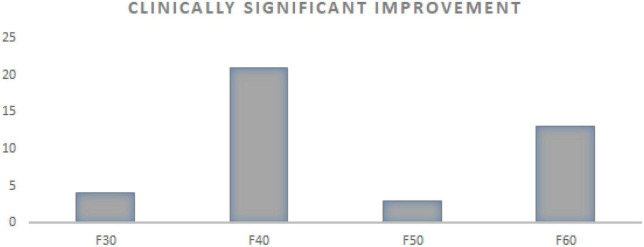
Number of patients with clinically significant improvement in relation to ICD diagnosis (total number 41/71 patients). F30 F30-F39-mood affective disorders (total number 8). F40 F40-F49-anxiety, dissociative, stress-related, somatoform, and other non-psychotic mental disorders (total number 37). F50 F50-F59-behavioral syndromes associated with physiological disturbances and physical factors (total number 6). F60 F60-F69-disorders of adult personality and behavior (total number 20).

The results showed significant clinical changes among 50% of patients in F30−F39, 57% in F40−F49, 50% in F50−F59, and 65% in F60−F69 (see Figure 2).

## 4. Discussion

Our study is the first step in a process to verify the efficacy of a short-term integrated psychotherapy treatment approach using the FBIM model. We used four measures to test its effectiveness: (a) individual wellbeing; (b) problems/symptoms; (c) life functioning; and (d) suicidal risk before and after the treatment according to CORE-OM. Our results highlighted significant positive changes in all the subscales. In short, the participants appeared to benefit from the therapy. Our results are consistent with previous studies (Beutler et al., 2012; Miscioscia et al., 2018) that have noted the effectiveness of integrated psychotherapy. The FBIM follows this tradition by proposing an approach that integrates a psychodynamic reading of the patient’s functioning. Moreover, the FBIM model uses the life cycle filter (Gislon, 2000, 2005) to understand the developmental pressures that have contributed to the patient’s condition. According to this perspective, the patient begins to experience an intrapsychic conflict between their needs and their fear of moving forward. They then begin to implement dysfunctional defenses that often develop into symptoms. The FBIM focuses on the central intrapsychic conflict underpinning the patient’s state.

That significant differences were seen in each domain measured by the CORE-OM suggests that the FBIM focus on the most problematic mental dynamic for the patient led to direct benefits across the various life domains. However, we cannot infer a change in the deeper mental dynamics of the patient because the follow-up time of approximately 38 weeks was relatively short. Future studies involving longer follow-up times would improve our understanding of patient symptomatology and the efficacy of the model from a public health perspective.

The results also indicate that the FBIM may work independently of the therapist’s characteristics and personality, given that the study involved a large cohort of therapists of different ages and genders. It would be interesting to see whether the same might apply to higher case numbers (Dazzi et al., 2006).

We found a clinically significant improvement in more than 50% of the sample for each ICD group, which suggests that the changes were transversal to the type of nosographic and functional diagnosis and independent of the focal internal conflict or the kind of symptom developed. The FBIM approach could, therefore, be applied to the maximum range of patients experiencing conflict. In terms of the life cycle, however, the study demonstrated the FBIM’s effectiveness in only a part of the sample (i.e., those approximately 35 years of age).

The pilot study has some limitations. First, it was not possible to analyze the causal association between the CORE-OM domains and FBIM interventions directly. However, the results may prove useful for future prospective studies that test hypotheses on the efficacy of the FBIM approach, particularly among older populations, because each phase of the life cycle has its specific intervention. It would also be interesting to replicate the study using a larger sample of age groups and nosographic diagnoses. The FBIM works on patients with a mental conflict, but it would be useful to evaluate its effect on those with predominantly impaired functioning or with other foci.

Qualitative studies on the fear component of conflict might be conducted because this is the factor that generates conflict and blocks the execution of evolutionary tasks. More demographic data (e.g., education, work, and family characteristics) could be included to clarify its role and significance. In addition, further research into resilience would help us understand how far a patient’s premorbid characteristics impact the efficacy or duration of therapy.

Another limitation of the study is its monocentric study design and the absence of a real control group. The five non-clinical participants were too few in number to be considered a non-clinical sample; they were also help-seekers who exhibited psychopathological features, though these were under the cut-off limits expressed by the CORE-OM reference values. Given these concerns, the FBIM might be used in a higher number of cases where the therapists involved worked in different centers. We prefer simply to highlight the preliminary/explorative perspectives of our research rather than draw any inferences from the results.

## 5. Conclusion

In conclusion, this pilot study shows that the FBIM generated reliable change in both symptomatology and relationship issues among the participants. The results, therefore, provide evidence of the validity of integrated psychotherapeutic models that use both psychodynamic and cognitive techniques. Further studies are needed to validate the FBIM model.

## Data availability statement

The raw data supporting the conclusions of this article will be made available by the authors, without undue reservation.

## Ethics statement

The studies involving human participants were reviewed and approved by the Istituto per lo Studio e la Ricerca sui Disturbi Psichici (ISeRDiP). The patients/participants provided their written informed consent to participate in this study.

## Author contributions

All authors contributed to the design of the study, organized and analyzed the database, and wrote the different sections of the manuscript and contributed to the manuscript revision, read, and approved the submitted version.
